# Simulation Promotes Nurse Practice Readiness in Age-Friendly Health System Care

**DOI:** 10.1093/geroni/igaf122.1634

**Published:** 2025-12-31

**Authors:** Eleanor McConnell, Dana Convoy, Valerie Sabol, Nicole Blodgett

**Affiliations:** Duke University, Durham, North Carolina, United States; Duke University, Durham, North Carolina, United States; Duke University, Durham, North Carolina, United States; Duke University, Durham, North Carolina, United States

## Abstract

As the Age-Friendly Health System (AFHS) framework becomes the accepted standard in caring for older adults across the United States, health professions schools seek effective approaches to learning so their graduates are competent to provide care consistent with AFHS principles. The Duke University Nurses Advancing Geriatric Care Excellence (NU-AGE) Simulation program offers an innovative approach to preparing pre-licensure and advanced practice nursing students to care for older adults using simulations focused on assessment and management of challenges associated with mentation, mobility, medications, and what matters most to older adults. We developed, deployed, and evaluated a combination of commercially-available simulations using the Embodied LabsTM virtual reality platform, combined with simulations developed by our team. The new simulations were enacted using videotaped simulated patients, using professional actors. These cases were deployed using the hologram technology. Student experience and clinical performance were evaluated with 20 students, (15 pre-licensure RN, 5 nurse practitioner students). Students rated the experience very positively, with mean +/- SD of 99 +/- 1.2 and 98.1 +/- 4.1 for the virtual reality and hologram simulations respectively. Themes from open-ended evaluation responses included the value of an empathy experience for cognitive and sensory impairments, and opportunity to experience diagnostic dilemmas and how best to respond prior to going into a clinical rotation. Simulation education using advanced technology platforms represent a promising, innovative approach to preparing pre-licensure and advanced practice nursing students to mastering the competencies needed to practice within the AFHS framework upon graduation.

